# Mesenchymal Hamartoma: Prenatal and Postnatal Diagnosis by Imaging

**DOI:** 10.1155/2012/954241

**Published:** 2012-12-05

**Authors:** Alicia Martínez-Varea, Jose María Vila-Vives, Juan José Hidalgo-Mora, Antonio Abad-Carrascosa, Roberto Llorens-Salvador, Alfredo Perales-Marín

**Affiliations:** ^1^Department of Obstetrics and Gynecology, La Fe University Hospital, 46026 Valencia, Spain; ^2^Department of Radiology, La Fe University Hospital, 46026 Valencia, Spain

## Abstract

We present a case of a twin pregnancy in which one fetus developed a rapidly growing unilateral intrathoracic tumor. While a cystic adenomatoid malformation was suspected in the ultrasound scan, the magnetic resonance scan suggested a pulmonary blastoma or a bronchioalveolar carcinoma. Postnatal chest radiography and contrast-enhanced computed tomography of the affected newborn were performed, and it was ruled out the possibility of malignant origin. Finally, the anatomopathologic exam revealed the presence of a mesenchymal hamartoma in the chest wall. Nevertheless, parents refused any treatment for the newborn.

## 1. Introduction 

Fetal tumors are heterogeneous group of neoplasias that are unique as far as their histological characteristics, anatomical distribution, and physiopathology are concerned. The biological behavior of tumors in the fetus may differ drastically if compared with an adult [1]. 

Congenital thoracic malformations, which comprise a wide spectrum of abnormalities, are often detected by routine prenatal ultrasound. However, the diagnosis usually cannot be established with absolute certainty before birth. Their presence involves having to rule out if there is a pulmonary issue or if its origin lies in the costal wall and does not affect the lung since the diagnosis impression, the prognosis, and treatment differ [2]. Ultrasound scans and, more recently, fetal magnetic resonance allow us to locate, delimit, and characterize the lesion type in certain cases [3].

Mesenchymal hamartomata in the chest wall are extremely infrequent benign tumors that tend to appear during infancy, and their presence in the prenatal period is exceptionally rare [4]. Clinically, they vary from asymptomatic to severe respiratory distress which requires intubation. The definite diagnosis is anatomopathological, and thus prenatal imaging techniques help confirm suspicion and propose the best postdelivery treatment [3, 5]. 

The congenital cystic adenomatoid malformation of the lung is a rare fetal affection with an incidence of 1 : 25000 to 1 : 35000 pregnancies [6]. When a unilateral anechoic thoracic lesion is discovered, any cystic adenomatoid malformation (CAM) must be ruled out: type 1 (cysts with a shiny surrounding measuring 2–10 cm) or type II (between 0.5–2 cm) [6]. Three CAM types are distinguished, and a type III CAM corresponds to the ultrasound scan image of a unilateral hyperechoic lesion comprising cysts less than 0.5 centimeters. 

We present a case of a twin pregnancy with one fetus with a rapidly growing unilateral intrathoracic tumor. From the ultrasound scan, a cystic adenomatoid malformation was suspected, while the magnetic resonance scan suggested a pulmonary blastoma or a bronchioalveolar carcinoma. Finally, the anatomopathologic exam revealed the presence of a mesenchymal hamartoma in the chest wall. The literature includes very few cases of mesenchymal hamartoma with accelerated growth complicated with hydrops fetalis in one twin during a bichorial biamniotic twin pregnancy. 

## 2. Case Report

**Figure 1:**
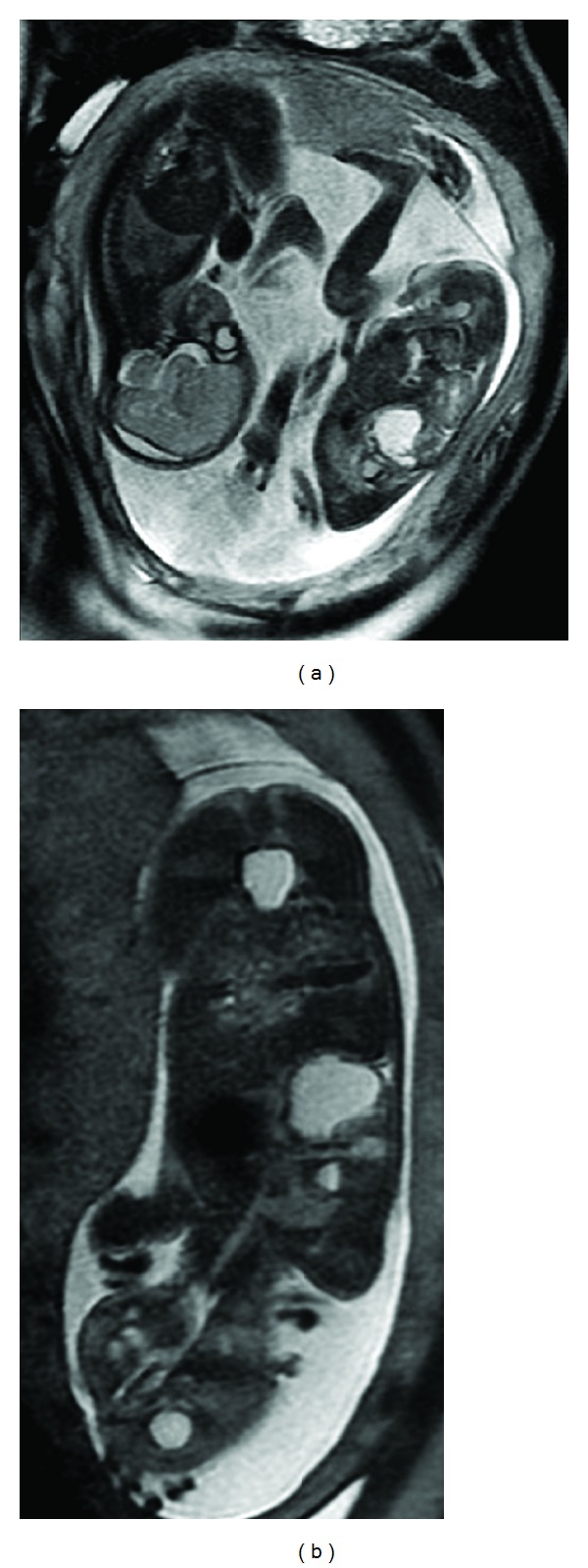
The MR revealed a lesion with several cysts (hyperintensive at T2) occupying a space in the right inferior lobe. A more caudal cyst, and a second cranial cyst with a liquid level were noted.

A 31-year-old nulliparous woman presented a bichorial biamniotic twin pregnancy, accomplished by artificial insemination due to primary sterility problems. The patient had no outstanding medical or surgical background. The fetal morphology ultrasound scan, performed at 20-week gestation, showed that both fetuses were apparently normal. Pregnancy developed normally until 23 weeks of gestation. An ultrasound scan showed supradiaphragmatic avascular cystic formation in the right hemithorax of the first fetus of 34 × 26 mm, suggesting a type I cystic adenomatoid malformation (CAM). Serial ultrasounds were performed by the maternal-fetal medicine service every two weeks, starting at 23 weeks, 3 days of gestation. As part of the diagnostic imaging protocol, fetal magnetic resonance (MR) was performed at 23 + 5 weeks of gestation. Sagital, coronal and axial fetal MR revealed a lesion with several cysts (hyperintensive at T2) occupying a space in the right inferior lobe. A more caudal cyst was noted, measuring 25 × 20 mm, with a second cranial cyst of 11 × 22 mm with a liquid level (Figure 1). 

Given the discovery of more thickened cystic walls than usually found in a CAM, and since the chest wall was apparently affected, the radiologist suggested considering a pulmonary blastoma or bronchioalveolar carcinoma, tumors described in patients with a previous CAM. However, since CAM-associated tumors have been described to appear postnatally and given the ultrasound scan findings, the first diagnostic suspicion was a type I CAM in the first twin. The amniocentesis at 25 + 1 weeks of gestation proved normal XY karyotypes for both fetuses. The ultrasound scan done at the time of this invasive technique showed an image that was compatible with a CAM in the first fetus' right hemithorax. A subsequent ultrasound scan at 28 weeks of gestation evidenced moderate ascites and hydrops fetalis. 

At 30 + 5 weeks of gestation, despite tocolytic treatment to prevent preterm delivery, a cesarean section had to be performed because of podalic presentation of the first fetus. The first male newborn weigh was 1700 g, Apgar score 8/10, umbilical arterial pH (ApH) 7.27 (mean 7.28, SD 0.05), umbilical venous pH (VpH) 7.39 (mean 7.35, SD 0.05), and he needed assisted ventilation. Second twin weighed 1400 g, Apgar score 9/10, ApH 7.28, VpH 7.36, and a favourable outcome. 

**Figure 2:**
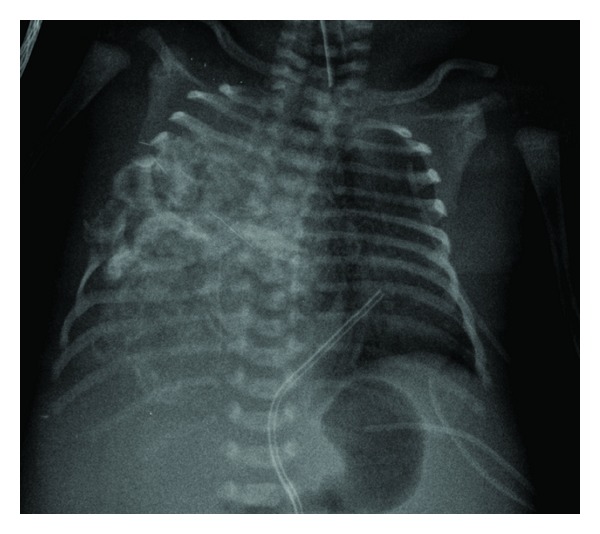
Chest X-ray showed right hemithorax opacification due to a large-sized mass with calcium density and destruction/erosion of the 4th–7th right costal arches, plus leftward tracheal deviation and centralized intestinal luminogram.

**Figure 3:**
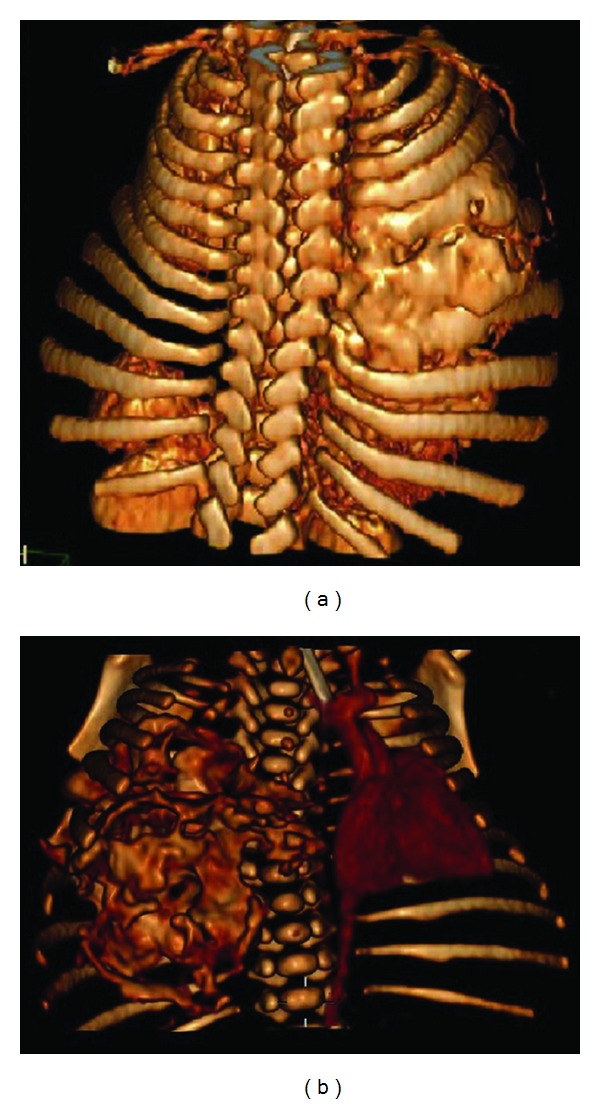
Posterior and anterior volume-rendered computerized axial tomography with contrast injection revealed a mass occupying the right hemithorax measuring 60 × 50 × 50 mm (the cranial-caudal, transverse, and anteroposterior diameter) in contact with the right costal wall which it appeared to depend on.

Postnatal chest X-ray revealed right hemithorax opacification due to a large-sized mass with calcium density and destruction/erosion of the 4th–7th right costal arches, plus leftward tracheal deviation and centralized intestinal luminogram, probably because of ascites (Figure 2). A contrast-enhanced computed tomography of one newborn was obtained. That was elegantly demonstrated there a mass occupying the right hemithorax measuring 60 × 50 × 50 mm, in contact with the right costal wall which it appeared to depend on (Figures 3(a) and 3(b)). Pediatricians explained to the parents that the suspected congenital tumor of the newborn could be a mesenchymal hamartoma. They also explicated to them the treatment and prognosis. But parents refused any therapeutic option for the newborn child who died a few minutes after withdrawing the endotracheal tube. 

The mother had an uncomplicated immediate puerperium and was discharged 72 hours after the cesarean section. Mesenchymal hamartoma of the newborn was confirmed by anatomopathological study made after the autopsy. 

## 3. Discussion

Congenital thoracic malformations are often detected by prenatal ultrasound. Their presence involves having to rule out if there is a pulmonary issue or if its origin lies in the costal wall and does not affect the lung because the prognosis and treatment differ [2]. Ultrasound scans and fetal magnetic resonance allow us to locate, delimit, and characterize the lesion type in certain cases [3].

Fetal thoracic malformations are examined by color Doppler images and nuclear magnetic resonance (NMR) scans, and the perinatal anatomopathological study proves definitive to associate tumors. The NMR scan allowed a better definition of the main characteristics than the ultrasound scan and also a better spatial valuation with orthogonal tridimensional views [3]. When a unilateral anechoic thoracic lesion is encountered, it is necessary to rule out a type I cystic adenomatoid malformation (CAM) (cysts with a shiny surrounding measuring 2–10 cm) or a type II CAM (between 0.5 and 2 cm), left congenital diaphragmatic hernia (Bochdaleck), unilateral hydrothorax, or a bronchogenic cyst, among others. The characteristics of the fetal tumor in the present case report were compatible with a type I CAM. The incidence of CAMs is 1 : 5000 live-born neonates [7].

We present a case of a twin pregnancy with one fetus with a rapidly growing unilateral intrathoracic tumor. From the ultrasound scan, a cystic adenomatoid malformation was suspected. A NMR scan was done at 23 + 5 weeks gestation, which revealed a lesion with several cysts (hyperintensive at T2) occupying a space in the right inferior lobe. A more caudal cyst was noted, measuring 25 × 20 mm, with a second cranial cyst of 11 × 22 mm with a liquid level (Figure 2). There was a third larger lateral cyst measuring 18 × 15 mm, which appeared to be located in the chest wall. A contralateral deviation of the mediastinum was evident. Given the discovery of more thickened cystic walls than usually found in a CAM, and since the chest wall was apparently affected, the radiologist suggested considering a pulmonary blastoma or bronchioalveolar carcinoma, tumors described in patients with a previous CAM. Finally, the anatomopathologic exam revealed the presence of a mesenchymal hamartoma in the chest wall.

Mesenchymal hamartomata in the chest wall are extremely infrequent benign tumors that tend to appear during early infancy, and their presence in the prenatal period is exceptionally rare [4]. They infrequently develop in late infancy, and the oldest age at diagnosis reported is 12 years [4]. Clinically, they vary from asymptomatic to severe respiratory distress which requires intubation. The definite diagnosis is anatomopathological, and thus prenatal and postnatal imaging techniques help confirm suspicion and propose the best post-delivery treatment [5, 7]. In recent years, ultrasound scanning and magnetic resonance imaging have helped identify more perinatal thoracic hamartomata. Nonetheless, an ultrasound scanning finding of the case reported herein and the infrequent incidence of perinatal hamartomata suggest a preliminary suspected diagnosis of type I CAM. 

Mesenchymal hamartomata are frequently located to the rear, tend to affect many adjacent ribs, and may be multifocal and bilateral. Hamartomata in the bilateral chest wall are an infrequent occurrence and tend to be confused with a malignant lesion. They may involve distortion and cardiac and lung displacement [4]. In ultrasound scans, they appear as heterogeneous intrathoracic masses. They may be associated with massive pleural bleeding which requires fetal pleuro-amniotic shunts. In radiological terms, they are shown as expansive costal masses with an associated extrapleural mass of soft tissues [8]. Costal molding, erosion, and expansion denote a slow-growing process. Some hemorrhagic cavities may form with secondary liquid-liquid levels to aneurismatic bone cysts formation. These liquid-liquid levels are better visualized in cross-sections and can be more easily visualized by NRM than by CAT. The NRM images at T1 and T2 reveal a heterogeneous intensity signal. Areas showing hyperintensity at T1 reflect hemorrhaging [8]. 

A correct mesenchymal hamartoma diagnosis is crucial because most masses located in the chest wall of infants are malignant. As previously mentioned, a biopsy provides a definite diagnosis. Macroscopically, the hamartoma has a bloody appearance and contains small cysts. Microscopically, mature adipose tissue mixed with a large number of veins with various diameters and with collagen tissue is observed, and primitive mesenchymal elements with groups of lymphoid cells can be focally visualized [5]. Histologically, these lesions are composed of heterogeneous components of a mesenchymal origin, which can be easily misinterpreted as being malignant given their proliferative appearance and cellular irregularity [7]. 

Several forms of treatment have been described, including the surgical excision of the mass and thermal radioablation. It is believed that the hamartoma becomes smaller in size after the first two years of life [5]. Therefore, conservative treatment of asymptomatic children is feasible in centers which have suitable diagnostic imaging techniques capable of regularly monitoring the patient, and which offer surgical intervention if required [4]. Should the lesion grow during the first two years of life, or should tracheal or lung compression be detected, surgical removal of the tumor is recommended. The surgical procedure for asymptomatic lesions consists in a resection en bloc, and the resulting defect in the chest wall can be reconstructed by prosthetic meshes or by a muscular flap [7, 8]. 

In conclusion, mesenchymal hamartomata have to be beared in mind in the differential diagnosis of fetal thoracic lesions. The encouraging prognosis of this benign tumour should be carefully reported to parents.
